# The development and evaluation of a holistic needs assessment and care planning learning package targeted at cancer nurses in the UK

**DOI:** 10.3332/ecancer.2014.416

**Published:** 2014-04-10

**Authors:** R Henry, B Hartley, M Simpson, N Doyle

**Affiliations:** Living With and Beyond Cancer Forum, The United Kingdom Oncology Nursing Society (UKONS), London, UK

**Keywords:** holistic needs assessment (HNA) and care planning, elearning, cancer nursing, survivorship, living with and beyond cancer

## Abstract

A project team from the United Kingdom Oncology Nursing Society developed a blended e-learning website to facilitate nurses to further develop their confidence and competencies in a range of skills related to assessing the holistic needs of people with cancer. The project team identified three areas which were integral to an holistic needs assessment (HNA) implementation project. These were project support information, project management skills, and practical competencies delivered in a blended e-learning package containing a series of accessible video presentations, supporting documents, and practical activities. The team worked with internal and external partners to ensure that a robust and inspiring programme was created.

www.hnaforcancer.com was launched in October 2012 as a blended learning programme that incorporates e-learning on core subjects. These subjects are packaged as videoed presentations with supporting learning material and can be accessed via the UKONS website. By the end of the programme participants were equipped to identify and explore the essential requirements for HNA and care planning, more able to recognise potential need, and initiate care to prevent or minimise the risk of complications. Participants had also developed confidence and competency in new skills, including basic project management.

## Introduction

A diagnosis of cancer and its subsequent treatment may result in continuing physical, emotional, psychological, and spiritual distress that negatively impacts quality of life [1–3]. Trends demonstrate that increasing numbers of people are living with their diagnosis of cancer and beyond their experience of active treatment. Their needs are widely acknowledged throughout the United Kingdom, notably in England’s Cancer Reform Strategy [4] and Scotland’s Better Cancer Care [5] with Wales’ Cancer Services Coordinating Group and Northern Ireland’s Transforming Cancer Follow-up (TCFU) continuing projects in this area.

However, despite the widespread acceptance of the needs of those living with and beyond cancer, the evidence suggests that health-care professionals often fail to detect the extent of distress in cancer patients [6–10]. Paradoxically, many people report that the intensity of support from health-care professionals actually tends to decline as treatment ends [11] suggesting that those with inadequately addressed concerns and unrecognised issues fail to get the multidisciplinary support they need and that this results in poorer overall health and dissatisfaction [12].

Consequently, it is axiomatic that a more formal structure for improving psychological outcomes for people living with and beyond cancer is needed to help health-care professionals identify the incidence and prevalence of distress [13]. The Cancer Reform Strategy [4] and [14] recommend that those living with and beyond cancer should have their holistic needs addressed whilst the National Cancer Action Team (NCAT) [15] identified the importance of developing patient needs assessment approaches and tools for use in clinical practice.

The National Cancer Survivorship Initiative define a holistic needs assessment (HNA) as, ‘a process of gathering information from the patient and/or carer to inform discussion and develop a deeper understanding of what the person understands and needs’ and is concerned with the whole person by incorporating their physical, emotional, spiritual, social, and environmental well-being [14].

## HNA and care planning learning package

In accordance with the aims of the United Kingdom Oncology Society (UKONS) and in response to ongoing discussion among Society members about the implementation of HNA in clinical practice, the UKONS Board agreed to support the development of a learning package on this subject. It was agreed that the key features of any package were that it should be readily accessible to UKONS members, that it should incur no personal cost to individual members and that it should be appropriate and applicable in all four countries of the United Kingdom. Additionally, it was accepted that those undertaking the course should receive some kind of acknowledgment/certification on completion of the package, and whilst the certificates cannot be traded for academic credit, they are indicative of ongoing personal and professional development. It was accepted that, whilst the course should be designed primarily for UKONS members, it ought to be freely available to any health-care professional involved in the care of people with cancer.

The twin aims of this project were clarified. These were, first, to help cancer nurses to develop their understanding of the concept of HNA and its application in clinical practice. Second, the project should focus on identifying and nurturing the confidence as well as the competence and team working skills needed to implement HNA into clinical practice. It was accepted that, for many cancer nurses, this might mean instigating and leading change in practice. Consequently, the programme also addressed the need to empower and enable cancer nurses to introduce and lead change at a local level.

In response to this specification, in early 2012 a Project Team from the UKONS developed an e-learning package focusing on HNA. This incorporated a series of video presentations, supporting literature, and practical activities in a dedicated website, www.hnaforcancer.com. It was agreed, early in the project, that an e-learning format was the most appropriate way of meeting the specifications outlined above. McVeigh [16] found the benefits of e-learning to lie in its flexibility in time management, pace of learning, self-direction, and wide access to information. However, she cautions that factors such as computer literacy, lack of work-based support, perceptions of e-learning, and competing home pressures may adversely affect the educational experience.

The discussion within the Project Team about the potential of an e-learning approach is encapsulated (Table 1), and, consequently, it was agreed that an evaluative mechanism should be integral to the project.

The programme consists of five modules of online learning, each of which feature online video presentations and links to supporting literature and allied study material (Table 2). Each module contains video presentations timed and paced in a way that is designed to suit practising clinicians. To accompany each presentation, learners are provided with downloadable study material including a step-by-step project stage guide complete with background information. Study material also includes activity ideas, plus all the tools needed to complete these activities. Additionally, there are suggested follow-up activities to assist with the retention of key points, and, finally, learners will also be provided with materials to assist in patient education and communication with clients.

Each module is designed to take approximately 1 h to complete; after that, participants are directed to an online multiple-choice scenario-based questionnaire in which participants can consolidate and assess their personal learning. On completion of all modules, participants will be awarded a Certificate of Completion. At this point, ongoing support is offered through contact with the UKONS Living With and Beyond Cancer Forum.

Whilst there are obvious advantages and disadvantages to e-learning, flexibility and ease of access were regarded as prime considerations in the design of the programme. Thus, the programme contains the ability to log-in and log-out of the programme in a timeframe that suits cancer nurses.

## Key deliverables

On completion of the programme, it is intended that participants should be introduced to the range of knowledge, understanding, and skills that are integral to HNA and care planning. This is underpinned by an emphasis on effective communication with patients, carers, clients, and other relevant parties. This was deemed as important because, in helping participants to recognise the range and extent of the needs of those living with and beyond cancer, an emphasis is placed on their ability to educate and motivate others to similarly improve their awareness, learning, and behaviours regarding effective HNA and care planning. An understanding of basic project management is, therefore, seen as a key element of this course as cancer nurses actively engage in the development of their peers.

## Evaluation

The e-learning package was launched at the UKONS Annual Conference 2012 and marketed to all UKONS members. As planned, six months after its introduction, the same UKONS Project Team conducted an evaluation of the initiative. The evaluation process consisted of linking the responses from an on-line questionnaire with other relevant website data to gain an overall picture of the uptake and impact of the HNA and care planning e-learning package. The questionnaire consisted of Likert-type questions that explored pertinent issues, such as ease of access, value in enhancing nursing practice, usefulness of supporting material, and overall value of the package as a developmental tool. A section of the questionnaire was additionally assigned to allow respondents an opportunity to comment, in free text, on any other aspect of the programme.

Questionnaires were sent to all those who had completed the programme and applied for certification. Questionnaires were also posted on the UKONS website to allow those who has started, but not completed, the programme to comment upon its value. Responses to the questionnaire were anonymous, so it was impossible to ascertain the how many replies came from each source. A limited response of ten questionnaires was less than hoped but was arguably consistent with [17] the contention that email surveys are often associated with a poor response rate when compared with other approaches, such as postal surveys. However, they also suggest that response quality (including the number of questions answered, omissions, and response to open-ended questions) often attracts less attention than it merits and can provide useful data. In this respect, the survey together with the other website data produced enough valuable material to support reasonable conclusions about the HNA e-learning package.

## Results

Using website data, it was possible to ascertain that, over its lifetime, there were an average of 100 unique visits per month, which translated into a mean of 289 page visits per month, with 2.88 pages viewed per visit. The average duration of each visit was 4 min. Interestingly, whilst most visits were from the United Kingdom, there was uptake from other countries, notably Australia, with nurses from Ireland and Germany also showing an interest.

**Figure 1:**
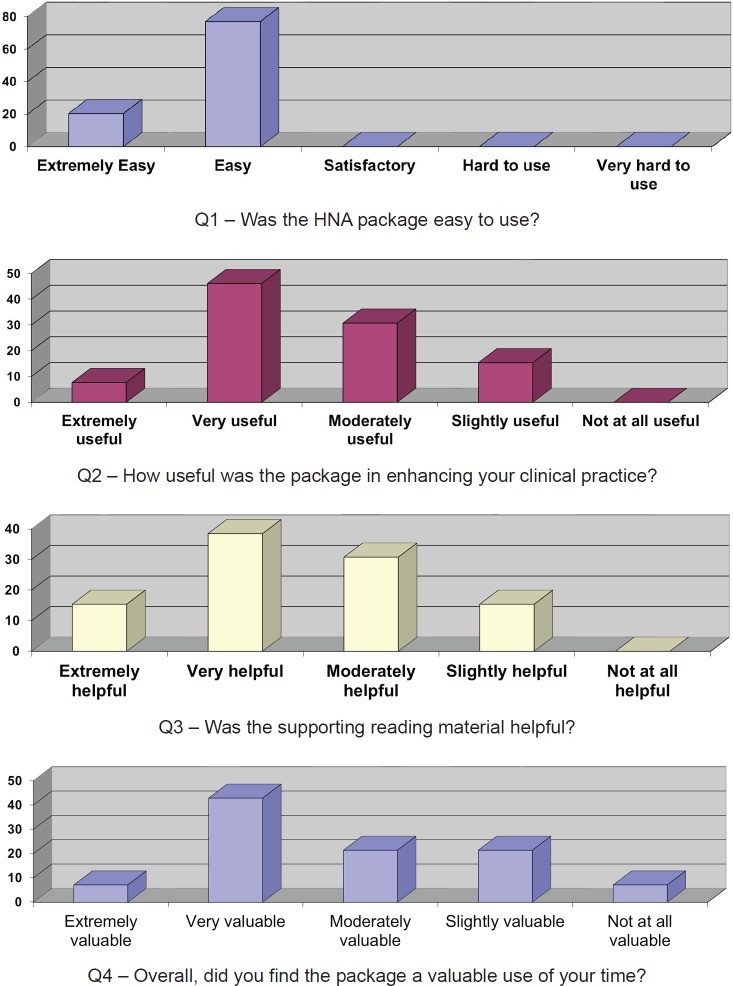
The questionnaire responses.

Details of the questionnaire responses are presented below (Figure 1) indicating that all respondents found the package easy or very easy to use. A respectable 53% found the package useful or very useful in enhancing clinical practice (with nobody commenting that they found the package without some value). The supporting reading material was regarded as helpful, although this ranged from moderately to extremely helpful. Overall, the package was regarded as valuable use of time and the majority of respondents (61%) would recommend the package to colleagues.

## Discussion

Whilst this feedback might be regarded as generally positive, it also indicates that there is room for improvement in the content and delivery of the e-learning package. Many of the suggestions for improvement were contained in the free text that respondents to the survey were encouraged to add whilst completing the questionnaire. Several responses indicated that they would have liked something longer and more in depth whilst others commented (both favourably and unfavourably) on the decision to marry the section on those skills and insights needed to conduct a HNA with the piece on change management. These were felt to be a reasonable and justified comments although the UKONS Project Team have always felt that cancer nurses should not just perform at a high level but also show the way for others to develop their practice.

For the UKONS Project Team, the decision to develop the HNA and care planning e-learning package was vindicated by the quite widespread uptake and the largely positive response. This approach represents an important method of engaging with the large and widespread cancer nursing community and offering them development opportunities and support that they may not otherwise be able to access.

The results of the evaluation were relayed to the UKONS Board, where they were acknowledged and discussed. The project was regarded as largely successful, and it was agreed that the programme should continue to be offered in its current format to UKONS members. Additionally, it may pave the way for similar initiatives in the future in other areas of cancer nursing practice.

## Figures and Tables

**Table 1:** Advantages/disadvantages of e-learning.

Advantages	Disadvantages
• Just-in-time learning	• Requires computer access
• Standardised learning	• Requires internet access
• Self-paced learning	• Requires basic computer skills
• Flexible means of assessment	• Does not suit all learners
• No travel costs	• Development costs of good-quality bespoke material can be high
• No classroom accommodation	
• Cost-effective	• Not all material may be compatible with underlying learning management system

**Table 2:** Module content.

Module
Module 1: HNA—Why?
Module 2: HNA for Teams—Planning
Module 3: HNA for Teams—Implementing
Module 4: HNA—What and How?
Module 5: HNA—Evaluating
